# Association of serum miR-99a level and metabolic dysfunction-associated steatotic liver disease, serum mTOR levels in patients with type 2 diabetes mellitus

**DOI:** 10.3389/fendo.2025.1724108

**Published:** 2025-12-03

**Authors:** Yangyang Zhang, Yuqiong Zuo, Qian Chen, Yaqiang Cui, Yanxia Bao, Panpan Jiang, Jing Liu, Jinxing Quan, Juxiang Liu

**Affiliations:** 1 Department of Endocrinology, Gansu Provincial Hospital, Lanzhou, Gansu, China; 2 Clinical Medical College, Ningxia Medical University, Yinchuan, Ningxia, China; 3 Medical Record Management Department, Gansu Provincial Hospital, Lanzhou, Gansu, China; 4 Key Laboratory of Endocrine and Metabolic Diseases of Gansu Province, Lanzhou, Gansu, China; 5 The First Clinical Medical College of Lanzhou University, Lanzhou, Gansu, China

**Keywords:** type diabetes mellitus, metabolic dysfunction-associated steatotic liver disease, miR-99a, mTOR, miRNA

## Abstract

**Purpose:**

This study was designed with the goal of exploring miR-99a expression in T2DM patients suffering from comorbid MASLD and clarifying the importance of miR-99a in this pathological context.

**Methods:**

A total of 137 subjects were included in this study, including 50 T2DM patients with MASLD (T2DM +MASLD group),48 T2DM patients without MASLD (T2DM group), and 39 healthy subjects (Control group). We measured the levels of IL-6, mTOR and SOD in the serum of the subjects by ELISA. The plasma miR-99a levels was detected by RT-PCR. The correlation between serum miR-99a level and other indicators was analyzed.

**Results:**

Serum miR-99a levels (median 0.79 vs 0.16 vs 0.03, *P* < 0.001) were significantly lower in the T2DM group than the healthy population and further decreased in the T2DM with MASLD patients (*P* < 0.001). After adjusting for age, gender, illness duration and BMI, spearman correlation analysis showed that TG, HbA1c, FPG, HOMA-IR, Hs-CRP, IL-6, HDL-C, mTOR(*P* < 0.05) remained independently linked with serum miR-99a. And stepwise linear regression analysis showed that HbA1c, IL-6 and mTOR are independent serum miR-99a correlation variables (*P* < 0.05). Moreover, the ROC results indicated that serum miR-99a has a high diagnostic value for T2DM with MASLD. In conclusion, serum miR-99a may be utilized as a screening biomarker for T2DM with MASLD.

**Conclusions:**

These data highlight a potential role for miR-99a as a regulator of the comorbid incidence of T2DM and MASLD, suggesting that measuring the levels of miR-99a can effectively predict the risk of MASLD in those with T2DM.

## Introduction

Type 2 diabetes mellitus (T2DM) is among the most common forms of chronic disease, and many T2DM patients suffer from comorbid metabolic dysfunction-associated steatotic liver disease (MASLD) (1), which impacts upwards of 70% of individuals with T2DM (2, 3). The risk of T2DM incidence is also approximately two-fold higher among patients with a MASLD diagnosis (4), and the alleviation of MASLD severity can modulate the risk of T2DM incidence in patients (5).

MicroRNAs (miRNAs) are 21–25 nucleotide-long transcripts that exhibit a high degree of evolutionary conservation and function through their ability to bind to target mRNA 3’-untranslated region (UTR) sequences, thereby suppressing translation or promoting degradation (6). Liver tissue reportedly exhibits high levels of miR-99a expression, while the downregulation of this miRNA has been reported in a range of cancers such as breast cancer (7), acute myeloid leukemia, and hepatocellular carcinoma, thereby promoting enhanced proliferative, invasive, and migratory activity in these malignant cells (8, 9). miR-99a-5p alleviates atherosclerosis through mTOR-mediated inhibition of NLRP3 inflammasome and promotion of macrophage autophagy (10). A significant correlative relationship between miR-99a downregulation in MASLD patient visceral adipose tissue and hepatic fibrosis (11). Experimental autoimmune encephalomyelitis (EAE) progression is alleviated by miR-99a, which functions as a therapeutic target by suppressing the mTOR signaling pathway and thereby modulating CD4+ T cell glycolysis and differentiation (12). Research points to the promise of combining spleen volume measurement with liver ultrasound as a low-cost and accessible new strategy, given the complex pathogenesis and diagnostic difficulties of early MASLD (13). As such, miR-99a may influence the incidence of severity of comorbid T2DM and MASLD, although no studies to date have specifically examined its role in this pathogenic context.

This study was thus developed to examine how serum miR-99a levels relate to the incidence of comorbid T2DM and MASLD through comparisons of samples from controls, T2DM patients, and T2DM with MASLD patients. Together, these analyses aim to define a novel target for treating and/or preventing T2DM and MASLD, offering a theoretical foundation for future interventional approaches.

## Materials and methods

### Research participants

For this study, we recruited 98 patients with T2DM from our hospital. Based on ultrasonography findings, they were stratified into two groups: 48 patients without MASLD and 50 patients with MASLD. In addition, 39 healthy individuals were selected as controls from the health examination center of our hospital during the same study interval. Subjects were excluded if they exhibited (1) viral hepatitis, hepatic cirrhosis, biliary tract obstruction, or autoimmune liver disease, (2) a history of alcohol use (> 210 g/week or > 140 g/week for over 12 months for males and females, respectively, (3) severe infections, acute diabetic complications, or pregnancy, or (4) were using drugs with the potential to impact lipid metabolism. The Clinical Research Ethics Committee of Hospital approved these studies, with all subjects providing written informed consent.

### Physiological and biochemical analyses

General physiological characteristics were recorded for all subjects, including age, sex, weight, height, Hip Circumference (HC), Waist Circumference (WC) and diabetes duration, enabling the calculation of both the waist-hip ratio (WHR; WC/HC) and BMI (weight/height squared (kg/m2)). Biochemical testing of parameters including TC, TG, LDL-C, HDL-C, FPG, DINS, HbA1c, and Hs-CRP was performed by the Laboratory Department of Gansu Provincial Hospital. The homeostatic insulin resistance (HOMA-IR) model was computed as follows: HOMA-IR=FPG×FINS/22.5. MASLD was detected based on carotid ultrasonography (7.5 MHz frequency color Doppler ultrasound, Siemens Acuson×300, Germany) performed by a trained sonographer. Standard testing procedures were used for all analyses.

### Analysis of serum miR-99a, mTOR, SOD, and IL-6 levels

Patient 5 mL venous blood samples were collected while fasting. These samples were centrifuged (3,000 rpm, 10 min, 4 °C), and serum was stored at -80 °C. To measure miR-99a levels, 200 µL of serum was combined with 600 µL of Trizol (Shanghai, China) to extract RNA at room temperature for 5 min, and 14 µL of DEPC H_2_O was used for dissolving RNA. Then, cDNA synthesis was performed with an All-in-One™ miRNA First-Strand cDNA Synthesis Kit 2.0 (Guangzhou, China). All qPCR reactions were performed in a 20 µL total volume with Taq Pro Universal SYBR qPCR Master Mix (Nanjing, China) and a LightCycler 480 instrument (Shanghai, China) with the settings: 95 °C for 3 min; 40 cycles of 90 °C 10 s, and 65 °C 30s. The 2^-△△CT^ method was used to assess relative gene expression. Plasma IL-6, SOD, and mTOR levels were detected with commercial ELISA kits (Shanghai, China) as directed, with standard curves being generated by plotting the concentrations of standards against absorbance at 450 nm, with logistic regression equations then being used to fit these data, enabling the calculation of sample concentrations.

### Statistical analysis

Data were analyzed with SPSS 27.0 (IBM, USA) and GraphPad Prism 9.4.1. Continuous normally distributed data are reported as means ± SD and analyzed with one-way ANOVAs, whereas non-normally distributed data were given in the form of medians with interquartile range ranges (M (P25, P75)) and compared with Kruskal-Wallis test. Categorical variables were reported as percentages. Relationships among variables were assessed with Spearman correlation test. Following adjustment for age, sex, BMI, and diabetes duration, a partial correlation method was used to assess these relationships. Univariate regression analyses were also employed to assess the interplay among different variables, after which a multiple regression model was established. Diagnostic performance was also evaluated with receiver operating characteristic (ROC) curves. *P* < 0.05 serves as the cut-off to define statistical significance.

## Results

**Figure 1:**
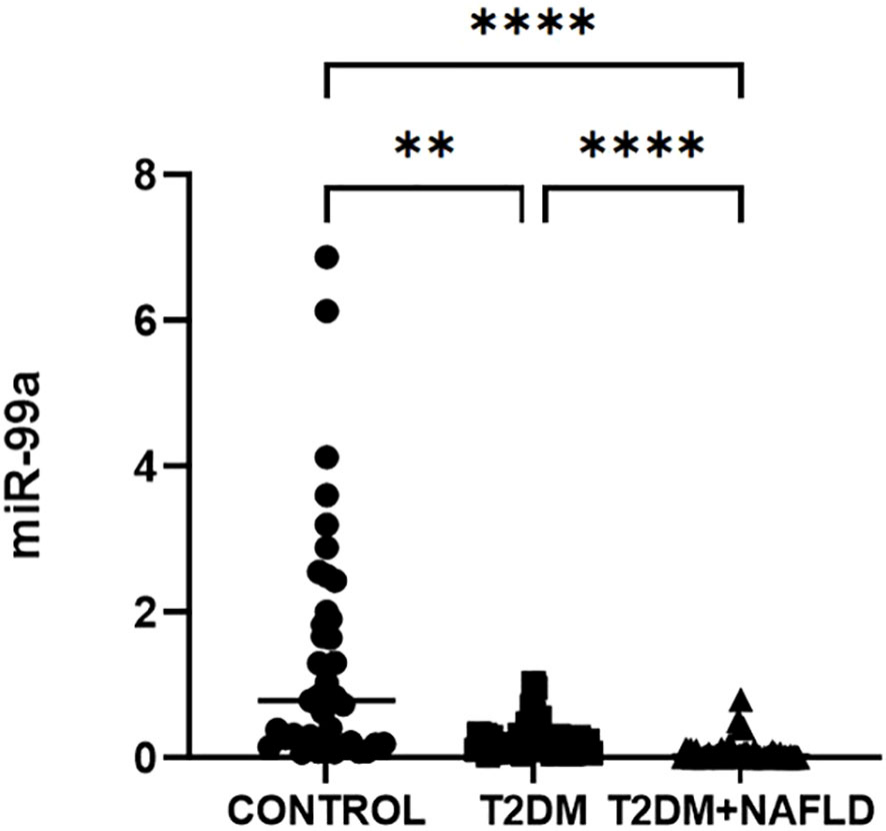
Serum miR-99a expression in the indicated participants groups. ****P<0.001; **P0.01.

Physiological and biochemical results from participants in the three subject groups are presented in **Table 1**. No differences in age, sex, BMI, LDL-C, or FINS were detected among these three groups, and diabetes duration was comparable in the T2DM and T2DM with MASLD (*P*>0.05). Compared with controls, the T2DM and T2DM with MASLD patients exhibited elevated IL-6, HbA1c, FPG, and HOMA-IR levels (*P* < 0.001), as well as reduced HDL-C and mTOR levels (*P* < 0.05), while these values did not differ when comparing T2DM patients with and without MASLD (*P*>0.05). Serum TG and Hs-CRP in T2DM+MASLD group were significantly elevated relative to control group and T2DM individuals (*P* < 0.001), whereas the opposite trend was observed with respect to SOD levels (*P* < 0.001), while in the control and T2DM groups were comparable (*P*>0.05). Significantly decreased median serum miR-99a levels were also noted in the T2DM group compared to healthy controls, with further reductions in T2DM+MASLD patients (0.79 vs 0.16 vs 0.03, *P* < 0.001) (**Figure 1**) (**Table 1**).

**Table 1:** Participant physicochemical characteristics.

Parameters	Control Group (n=39)	T2DM group (n=48)	T2DM+NAFLD group(n=50)	*P*
Age(years)	45.74±7.496c	48.27±5.414	47.84±5.947	0.224
Sex	22(56.4%)	27(56.3%)	28(56%)	0.999
Disease duration(years)	–	5(2,7)	3(1, 7.25)	0.435
BMI (kg/m2 )	24.39(23.24, 25.95)	23.856(21.78, 26.25)	25.40(23.57, 26.40)	0.128
WHR	0.919±0.047^c^	0.934±0.040	0.947±0.054^a^	0.029
TC (mmol/L)	4.39(3.87, 4.78)	4.17(3.20, 4.82)^c^	4.72(4.02, 5.63)^b^	0.022
TG (mmol/L)	1.23(0.96, 1.56)^c^	1.39(1.03, 2.17)^c^	2.23(1.53, 3.30)^a, b^	<0.001
HDL-C(mmol/L)	1.23±0.23^b, c^	0.98±0.24^a^	0.90±0.17^a^	<0.001
LDL-C(mmol/L)	2.61±0.48	2.57±1.04	2.89±0.74	0.045
HbA1c (%)	5.5(5.3, 5.8)^b, c^	8.4(7.7, 10.78)^a^	9.75(8.45, 11.46)^a^	<0.001
FPG (mmol/L)	5.13(4.86, 5.73)^b, c^	9.03(7.15, 12.86)^a^	10(7.35, 13.16)^a^	<0.001
FINS (mU/mL)	6.1(5.0, 8.7)	7.05(4.02, 10.70)	8.2(5.6, 12.03)	0.068
HOMA-IR	1.49(1.06, 2.12)^b, c^	2.72(2.06, 4.21)^a^	3.66(2.51, 5.81)^a^	<0.001
Hs-CRP (mg/L)	0.60(0.40, 0.90)^c^	0.95(0.50, 1.75)^c^	1.65(0.80, 3.33)^a, b^	<0.001
IL-6 (pg/mL)	16.41(13.55, 22.24)^b, c^	32.01(30.02, 39.92)^a^	43.33(31.11, 52.59)^a^	<0.001
SOD(pg/mL)	238.14±69.33^c^	213.67±53.88^c^	187.39±41.50^a, b^	<0.001
mTOR(pg/mL)	344.03(222.13, 408.80)^b, c^	199.91(118.18, 214.51)^a^	137.75(86.47, 177.79)^a^	<0.001
miR-99a	0.79(0.20, 2.01)^b, c^	0.16(0.11, 0.31)^a, c^	0.03(0.01, 0.08)^a, b^	<0.001

a, *P*<0.05 vs. Control group; b, *P*<0.05 vs. T2DM group; c, *P*<0.05 vs. T2DM+NAFLD group.

### Correlations between serum miR-99a and physicochemical parameters

The levels of miR-99a in patient serum were significantly negatively correlated with TG, HbA1c, FPG, HOMA-IR, Hs-CRP, and IL-6 levels, whereas miR-99a levels were positively correlated with mTOR and HDL-C in this patient cohort (*P* < 0.01) (**Table 2**). Following adjustment for age, sex, BMI, and T2DM duration, an independent association between serum miR-99a levels and HOMA-IR, Hs-CRP, TG, HbA1c, IL-6, HDL-C, FPG, and mTOR levels remained evident (*P* < 0.05) (**Table 2**). The Durbin-Watson (D-W) value for the regression equation was 1.714, indicating the absence of any autocorrelative relationship among variables consistent with good model construction. The mean of the standardized residuals was 0, while the SD was 0.989, consistent with an approximately normal distribution. These results thus revealed that HbA1c, IL-6, and mTOR levels were independently correlated with patient serum miR-99a levels (*P* < 0.05) (**Table 3**).

**Table 2:** Correlations between serum miR-99a levels and other parameters.

Parameters	miR-99a (unadjusted)		miR-99a (age, sex, disease course, and BMI adjusted)	
	r value	*P*-value	r value	*P*-value
Age (years)	0.088	0.309	–	–
Sex	-0.009	0.914	–	–
Disease duration (years)	0.056	0.584	–	–
BMI (kg/m2 )	0.046	0.591	–	–
WHR	-0.122	0.192	-0.124	0.155
TC (mmol/L)	-0.120	0.164	-0.089	0.309
TG (mmol/L)	-0.330	<0.001	-0.183	0.034
HDL-C (mmol/L)	0.333	<0.001	0.223	0.010
LDL-C (mmol/L)	-0.166	0.179	-0.056	0.454
HbA1c (%)	-0.457	<0.001	-0.392	<0.001
FPG (mmol/L)	-0.400	<0.001	-0.299	<0.001
FINS (mU/mL)	-0.128	0.135	-0.154	0.076
HOMA-IR	-0.358	<0.001	-0.288	<0.001
Hs-CRP (mg/L)	-0.282	<0.001	-0.231	0.007
IL-6 (pg/mL)	-0.523	<0.001	-0.346	<0.001
SOD (pg/mL)	0.160	0.061	0.109	0.210
mTOR (pg/mL)	0.441	<0.001	0.348	<0.001

**Table 3:** Stepwise multiple linear regression analyses.

Independent variable	B	Beta	SE	t	*P*	VIF
(Constant)	1.262		0.399	3.166	0.002	
HbA1c	-0.076	-0.188	0.038	-2.024	0.045	1.493
IL-6	-0.014	-0.220	0.006	-2.537	0.011	1.265
mTOR	0.002	0.218	0.001	2.556	0.012	1.253

### Correlative relationships among clinicopathological variables in patients with T2DM and MASLD

**Figure 2:**
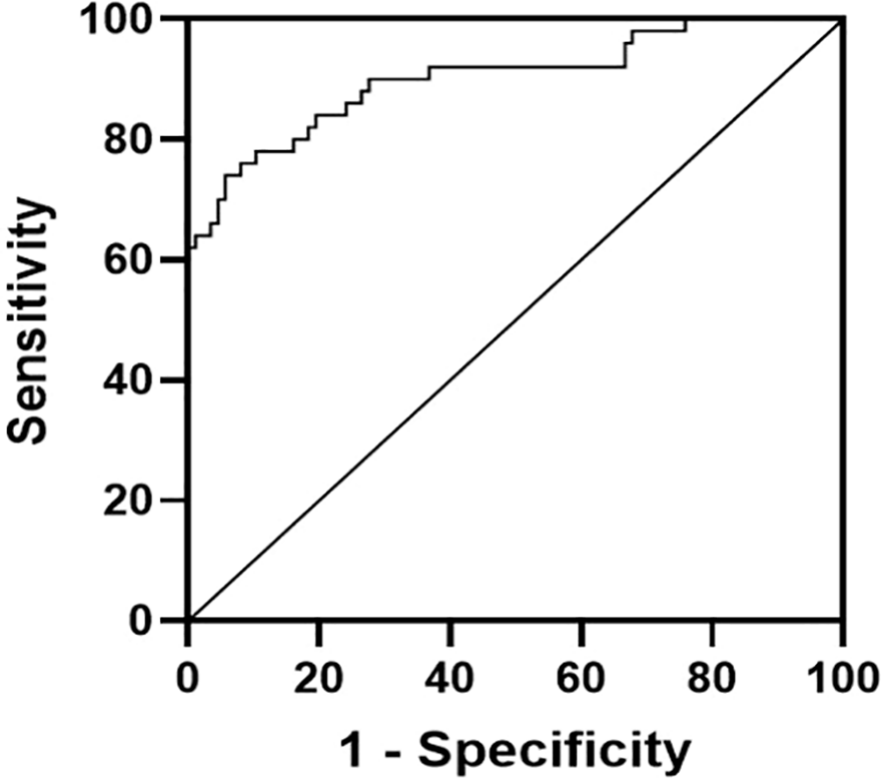
ROC curve.

Univariate analyses revealed TC, TG, HDL-C, LDL-C, HbA1c, FPG, FINS, HOMA-IR, Hs-CRP, IL-6, SOD, mTOR and miR-99a levels to be significantly associated with comorbid T2DM and MASLD (*P* < 0.05) (**Table 4**). With a cut-off value of 0.0678, the AUC was 0.9021 (95%CI: 0.8440-0.9601, *P* < 0.001), with respective sensitivity and specificity values of 94.3% and 76%, suggesting that serum miR-99a offers a high degree of diagnostic utility for T2DM with MASLD (**Figure 2**). Analyzing serum miR-99a may thus be an effective biomarker strategy when screening for comorbid T2DM with MASLD.

**Table 4:** Univariate logistic regression analyses of correlations between serum miR-99a levels and T2DM with NAFLD.

Parameters	OR	OR 95% CI	*P*-value
Age (years)	1.018	0.963-1.076	0.529
Sex	1.013	0.503-2.042	0.971
Disease duration (years)	0.972	0.865-1.092	0.630
BMI (kg/m2 )	1.134	0.974-1.320	0.106
TC (mmol/L)	1.599	1.117-2.289	0.010
TG (mmol/L)	2.308	1.526-3.490	<0.001
HDL-C (mmol/L)	0.019	0.003-0.136	<0.001
LDL-C (mmol/L)	1.609	1.018-2.542	0.042
HbA1c (%)	1.495	1.265-1.765	<0.001
FPG (mmol/L)	1.313	1.039-1.231	0.004
FINS (mU/mL)	1.106	1.019-1.201	0.016
HOMA-IR	1.575	1.277-1.944	<0.001
Hs-CRP (mg/L)	1.554	1.212-1.991	<0.001
IL-6 (pg/mL)	1.086	1.051-1.121	<0.001
SOD (pg/mL)	0.986	0.978-0.994	<0.001
mTOR (pg/mL)	0.989	0.985-0.994	<0.001
miR-99a	0.000	0.000-0.015	<0.001

All listed variables were considered for one-way logistic regression analyses. OR, odds ratio; CI, confidence interval.

## Discussion

MASLD represents a range of pathological conditions, including simple steatosis, metabolic dysfunction-associated steatohepatitis (MASH), and progression to fibrosis, cirrhosis and even hepatocellular carcinoma (HCC). The global prevalence of MASLD stands about 25%, with Asia reaching rates as high as 29.62% (14, 15).In one cross-sectional analysis of patients with T2DM from 20 nations, an estimated 55% of these individuals were also diagnosed with MASLD (16). The risk of T2DM is also approximately two-fold higher among MASLD patients (3). Excessive deposition of hepatic triglycerides triggers the activation of protein kinase Cϵ, leading to its translocation to the cell membrane, which hinders hepatic insulin signaling, resulting in decreased glycogen synthesis, increased gluconeogenesis, and fluctuations in insulin and glucose levels; on the other hand, the accumulation of ectopic fat in skeletal muscle contributes to muscle IR as skeletal muscle prefers *de novo* lipogenesis over glycogen synthesis, and furthermore, adipose tissue dysfunction, characterized by decreased adiponectin levels and increased long-chain fatty acids and pro-inflammatory cytokines, further exacerbates systemic IR (17). Specifically, T2DM promotes the progression of MASLD and accelerates the development of liver-related and extrahepatic adverse outcomes, while in turn, MASLD increases the likelihood of T2DM onset and exerts an adverse effect on glucose metabolism in the T2DM population (18, 19). Notably, the overlap between MASLD and T2DM not only increases the likelihood of liver-related adverse outcomes but also amplifies the risk of extrahepatic adverse outcomes, and cardiovascular disease (CVD) is the leading cause of death in populations with MASLD and T2DM (20).

In normal human hepatic tissue samples, miR-99a was identified as the 6^th^ most abundant miRNA, whereas it was significantly downregulated in visceral adipose tissue from MASLD patients and in individuals with HCC, with such downregulation being related to the incidence of hepatic fibrosis (11, 21). Here, T2DM patients were found to exhibit significantly decreased serum miR-99a levels compared to the control group, and these levels were lower still among individuals with comorbid MASLD (**Figure 1**). Li et al. reported an association between lower miR-99a levels and the insulin-inducible activation of mTOR. They observed that insulin treatment induced a two-fold decline in miR-99a expression in HL7702 cells, with the ability of insulin to modulate glycolytic activity being dependent on the inhibition of miR-99a expression such that the miR-99a/mTOR/HIF-1 axis plays a key role in shaping glucose consumption in response to insulin (22). There is evidence that miR-99a can also directly target mTOR (23). In one meta-analysis, Feng et al. found that mTOR is capable of directly regulating a range of inflammatory mediators including NF-κB and IL-6, protecting against MASLD development and progression. While the pathogenesis of MASLD is complex, many of the associated pathways are related to mTOR, as it is capable of indirectly and directly modulating autophagic activity and lipid accumulation within hepatocytes (24). Evidence also suggests that the anti-inflammatory action of miR-99a is mediated via a mechanism involving the negative regulation of glycolytic reprogramming in CD4+ T cells, achieved by targeting the mTOR pathway (12). PI3K/serine/AKT is an important signaling molecule downstream of IGF-1R, and phosphorylation of Akt regulates the expression of downstream target proteins such as mTOR and NF-κB, and plays an important role in signal transduction such as inflammatory response, glucose metabolism, and insulin resistance (25). The activator of the lipid synthesis signaling pathway on which PI3K/Akt depends is mTOR, and the abnormalities of the above signaling pathways affect the utilization of fatty acid synthesis substrates in hepatocytes, leading to the occurrence of metabolic inflammation, ultimately forming insulin resistance and promoting the development of nonalcoholic fatty liver disease (26). Here, significantly lower levels of both miR-99a and mTOR were detected in T2DM with MASLD patients compared to healthy subjects, and mTOR levels were independently related to miR-99a levels. As such, miR-99a may influence the combined progression of T2DM and MASLD via the mTOR pathway and related metabolic mechanisms.

Inflammation, oxidative stress, and IR all play roles in the onset of MASLD and T2DM (27–30). IR can contribute to a range of adverse metabolic outcomes such as hyperglycemia, dyslipidemia, prothrombotic state, visceral adiposity, inflammation, and dysregulated endothelial function that can lead to the development of these diseases. In patients with diabetes and prolonged hyperglycemia, abnormal levels of serum biomarkers of inflammation and oxidative stress including NF-κB and IL-6 are evident together with aberrant free fatty acid (FFA) metabolism. These changes contribute to functional alterations in hepatic interstitial cells and the hepatic microcirculatory system, impacting lipid metabolism and exchange between the blood and the liver in a way that promotes the incidence of MASLD (31). Here, T2DM patients diagnosed with MASLD presented with elevated serum HBAlc, FPG, IR, CRP, and IL-6 levels compared to HbA1c control subjects, with these correlations between these factors and miR-99a levels remaining evident even after controlling for a range of other factors. and IL-6 were both independently correlated with serum miR-99a in this patient cohort. Oxidative stress arises from the production of reactive oxygen species goes beyond the antioxidant system to mitigate associated damage, contributing to decreased peripheral insulin sensitivity and T2DM onset through various pathways. In individuals with MASLD, lower levels of antioxidant factors including coenzyme Q (CoQ), superoxide dismutase (SOD), and Cu-Zn SOD have been reported (32, 33). Relative to healthy controls, T2DM and T2DM with MASLD patients exhibited reduced serum SOD levels. In stepwise regression analyses, HbA1c and IL-6 levels were independently associated with miR-99a levels, suggesting that lower levels of this miR-99a may be related to IL-6 and HbA1c status, with all of these variables potentially shaping the onset or progression of T2DM with MASLD as a consequence of changes in miR-99a expression.

## Conclusion

T2DM patients were herein found to exhibit lower miR-99a levels than those in healthy controls, and these levels were even lower in patients with both T2DM and MASLD. A strong association between serum miR-99a and mTOR levels was also noted. Lower serum miR-99a levels may be independently associated with MASLD risk among T2DM patients, suggesting that it may be a valuable diagnostic or prognostic biomarker when screening for these comorbid diseases. Serum miR-99a levels are also functionally related to inflammation, IR, and glucolipid metabolism, underscoring the need for additional research focused on clarifying the pathophysiological role that miR-99a plays in the context of T2DM and MASLD.

## Data Availability

The raw data supporting the conclusions of this article will be made available by the authors, without undue reservation.
